# Epithelial–Fibroblast Crosstalk Protects against Acidosis-Induced Inflammatory and Fibrotic Alterations

**DOI:** 10.3390/biomedicines10030681

**Published:** 2022-03-16

**Authors:** Marie-Christin Schulz, Linda Voß, Gerald Schwerdt, Michael Gekle

**Affiliations:** Julius Bernstein Institute of Physiology, Magdeburger Straße 6, 06112 Halle, Germany; linda.wagenbrett@student.uni-halle.de (L.V.); gerald.schwerdt@medizin.uni-halle.de (G.S.); michael.gekle@uk-halle.de (M.G.)

**Keywords:** cellular crosstalk, chronic kidney diseases, extracellular acidosis, EMT, inflammation, fibrosis

## Abstract

Pathogenesis of chronic kidney disease (CKD) is accompanied by extracellular acidosis inflammation, fibrosis and epithelial-to-mesenchymal transition (EMT). The aim of this study was to assess the influence of acidosis on tubule epithelial cells (NRK-52E) and fibroblasts (NRK-49F) in dependence of cellular crosstalk. NRK-52E and NRK-49F were used in mono- and co-cultures, and were treated with acidic media (pH 6.0) for 48 h. The intracellular proteins were measured by Western blot. Secreted proteins were measured by ELISA. Distribution of E-cadherin was assessed by immunofluorescence and epithelial barrier function by FITC-dextran diffusion. Inflammation: Acidosis led to an increase in COX-2 in NRK-52E and TNF in NRK-49F in monoculture. In co-culture, this effect was reversed. EMT: Acidosis led to an increase in vimentin protein in both cell lines, whereas in co-culture, the effect was abolished. In NRK-52E, the E-cadherin expression was unchanged, but subcellular E-cadherin showed a disturbed distribution, and cellular barrier function was decreased. Fibrosis: Monoculture acidosis led to an increased secretion of collagen I and fibronectin in NRK-52E and collagen I in NRK-49F. In co-culture, the total collagen I secretion was unchanged, and fibronectin secretion was decreased. Intercellular crosstalk between epithelial cells and fibroblasts has a protective function regarding the development of acidosis-induced damage.

## 1. Introduction

The global prevalence of chronic kidney disease (CKD) is around 12%, leading to 1.2 million deaths annually [1,2]. Disturbance of the tubulointerstitial compartment, which creates a chronic inflammatory microenvironment, is an important factor for the chronic decline of renal function [3]. Thereby, inflammatory cytokines such as TNF and IL-6 as well as COX-2 metabolites appear in the tubule interstitial space [4,5,6,7], affecting the phenotype of epithelial cells and fibroblasts.

It is known that proximal tubule cells are often involved in the pathological events during the early stages of CKD [3]. This includes epithelial–mesenchymal transition (EMT) of proximal tubule cells [8,9]. EMT is characterized by increased expression of mesenchymal marker proteins such as α-smooth muscle actin (α-SMA) or vimentin. Moreover, epithelial marker proteins such as cadherin or occludin show decreased expression, besides enhanced degradation [10]. Additionally, the expression of transporter proteins, crucial for resorption and secretion, can be decreased, leading to impaired function [9,11]. Advanced states of chronic kidney disease are furthermore characterized by fibrosis [12]. This compromises the accumulation of fibrous matrix proteins, mainly collagen I and III, as well as fibronectin [13].

The main function of cells derived from the renal tubule is transepithelial transport. Epithelial cells from the proximal tubule as well as from the collecting duct are equipped with transporters, which allow them to secret protons by sodium proton exchange or by proton pumps, thereby playing an important role in pH homeostasis.

The main function of tissue fibroblasts is the secretion of matrix proteins, frequently fibrous collagens and fibronectin [14]. Although fibroblasts and epithelial cells maintain no direct contact, they can communicate via soluble mediators (e.g., cytokines and COX-2 metabolites) and matrix proteins [6,15]. The cellular crosstalk is involved in formation of the cytoskeleton, cell junctions, cell shape and matrix protein homeostasis. During the development of CKD, quiescent fibroblasts can be activated [12,14]. The changed phenotype is characterized by a larger nucleus, a higher proliferation rate, the expression of α-SMA and an increased secretion of cytokines, as well matrix proteins [16]. As a consequence, cellular crosstalk can change. Moreover, activated fibroblasts are involved in the development of an inflammatory and fibrotic milieu [14]. Subsequently, the resulting pathophysiological milieu can affect epithelial cells and can provoke an EMT.

Inflammation as well as fibrosis are accompanied by extracellular acidosis [17,18,19], whereby proximal tubule cells are exposed to pH values below 6.6. This extracellular acidosis is discussed as an additional stimulus promoting pathophysiological changes such as EMT, inflammation or fibrosis, resulting in a vicious cycle. 

In the present study, we investigated the impact of extracellular acidosis in a monoculture and in a co-culture model, consisting of rat proximal tubule cells (NRK-52E) and rat renal fibroblasts (NRK-49F). NRK-52E cells are well characterized and are described as a solid model for normal proximal tubule cells. This assessment is based on their epithelial characteristics, e.g., a flat polyhedral shape [20]. Furthermore, they dispose of cytoskeletal filaments and junctional proteins, which are typical for epithelial cells and which indicate a polarization of the cells [21,22]. Finally, studies have shown that NRK-52E cells express transport proteins that are characteristic for proximal tubule cells, e.g., OAT1, OAT3, SGLT2 and GLUT2 [23,24]. NRK-49F cells are described as normal fibroblasts. They are characterized by a spindle-like shape [20]. Moreover, they express mesenchymal marker proteins such as α-SMA and vimentin [25]. Based on these properties, these cell lines represent a suitable model to answer our question. The first question we addressed was if extracellular acidosis (media pH = 6.0) supports the development of inflammation, EMT and fibrosis in NRK-52E cells. The second question of this study was whether acidic media (pH 6.0) supports the activation of renal rat fibroblasts (NRK-49F). To approximate the in vivo situation better, we extended our experimental design to a co-culture model with NRK-52E and NRK-49F cells, using a filter insert, and enabling no direct contact except cellular crosstalk by soluble mediators. The co-culture enabled us to investigate the third question of this study, namely whether the cellular communication modulates the impact of extracellular acidosis. Again, we investigated the effects of acidosis and compared the acidosis impact in a mono- and co-cultures on both cell types.

## 2. Materials and Methods

### 2.1. Cell Culture

Normal rat kidney fibroblasts (NRK-49F, ATCC^®^, Manassas, VA, USA, CRL-1570) and normal rat kidney epithelial cells (NRK-52E ATCC^®^ ATCC^®^, Manassas, VA, USA CRL-1571) were grown in DMEM medium (Merck KGaA, Darmstadt, Germany, T041-10) supplemented with 5% fetal calf serum (FCS) (PAN-Biotech GmbH, Aidenbach, Germany, P30-3031) and 1.5 g/L NaHCO_3_ at 37 °C under a humidified 5% CO_2_ atmosphere and were subcultivated once per week before confluence.

### 2.2. Experimental Setup

For the experiments, NRK-52E cells were grown on permeable filter supports (pore size 0.4 µm) (Thermo Fisher Scientific, Waltham, MA, USA, 353,090 (6-well), 353,097 (24-Well), and NRK-49F cells were grown in 6-well plates to allow subsequent co-culture. After reaching full confluence, the cells were transferred to a medium without additional FCS supplementation for 24 h and were afterward incubated for up to 48 h at different pH values (adjusted with 1 N HCl, Carl Roth GmbH + Co. KG, Karlsruhe, Germany, K025.1). Control cells were exposed to pH 7.4. Extracellular pH (pHe) was measured with a blood gas analyzer (ABL5, Radiometer, Copenhagen, Denmark). Only a minor reduction in pHe of medium was observed during the chosen incubation periods. Therefore, the experiments could be performed under well-controlled conditions. Epithelial cells, such as NRK-52E-cells, attach to the substrate via their basolateral membrane, whereas the apical membrane faces toward the lumen. In vivo, this substrate is the basement membrane, which also separates the epithelial cells from the interstitial space that harbors fibroblasts. Thus, in order to mimic the in vivo situation properly, NRK-52E cells have to be cultured in the inserts and the fibroblasts in the well. The membrane of the insert represents the basement membrane [26].

### 2.3. Immunofluorescence

NRK-52E cells, cultured on coated glass coverslips, were treated with acidic media for 48 h and then fixed with 4% paraformaldehyde (Merck KGaA, Darmstadt, Germany, 1.04005) for 30 min. Afterward, the cells were incubated with 0.3% Triton X-100 (Sigma-Aldrich, Munich, Germany T-9284) for 10 min and blocked with 0.2% BSA (Capricorn Scientific GmbH, Ebsdorfergrund, Germany, BSA-FAF-1U) in PBS for 1 h. The primary anti-E-cadherin antibody (1:100) (see Table 1) was added and incubated for 1 h at room temperature. After three washing steps with 0.2% BSA in PBS, cells were incubated with Alexa Fluor^®^ 594-labeled secondary antibody (see Table 1). Subsequently, cells were incubated with 0.5 mg/mL DAPI (Molecular Probes, Eugene, OR, USA, D-1306) for 5 min. All images were obtained using a Keyence BZ-8100E-fluorescence microscope (Keyence Corporation, Osaka, Japan) at 40-fold magnification.

Afterward, cells were analyzed with the freeware ImageJ-win64 (https://imagej.nih.gov/ij/download.html, accessed on 17 May 2021). For the evaluation of E-cadherin distribution, maxima and minima values of the fluorescence intensity were assessed for both the cell membrane and the cytosol. Afterward, a mean for minima and maxima in the cell membrane as well as cytosol was calculated. Finally, a ratio between the value representative for the fluorescence intensity of the cytosol and of the total cell (cytosol + cell membrane) was calculated and multiplied by 100 to obtain the fraction of cytosolic E-cadherin [%]. Besides that, the membrane thickness and the circularity of the cells were calculated directly by ImageJ.

### 2.4. FITC-Dextran Diffusion

NRK-52E cells were seeded on the filter inserts (Falcon, Tewksbury, MA, USA). Then, 1.5 mL media was added to the apical compartment and 2.5 mL to the basolateral. After incubation with acidic media (pH 6.0), 1 g/L FITC-dextran (70 kDa) (Sigma-Aldrich, Munich, Germany, FD70S) was applied to the apical compartment, and after an additional 6, 24 and 48 h, 25 µL media aliquots were sampled from the basolateral and apical compartment. Before the measurement, the apical media were diluted 1:100 and the basolateral media were diluted 1:10,000 in HEPES-Ringer buffer. Fluorescence of FITC-Dextran was measured at 400 nm excitation and 505 nm emission wavelength using a multiwell reader (Infinite^®^ M200, Tecan, Crailsheim, Germany).

### 2.5. Collagen Direct ELISA

NRK-52E and NRK-49F cells in monoculture were seeded in 24-well plates or in co-culture as described in Section 2.3. The cells were incubated with media enriched with 50 mg/L ascorbic acid (Merck KGaA, Darmstadt, Germany, 127.250) to support collagen synthesis and 50 mg/L β-aminoproprionitrile (Sigma-Aldrich, Munich, Germany, A-3134) to avoid collagen polymerization, which improves collagen determination. Collagen and fibronectin contents were measured in the media according to and normalized to cellular protein content [27]. Antibodies against collagen I (see Table 1) was diluted 1:1000 and fibronectin 1 (see Table 1) was diluted 1:2000. HRP-coupled secondary antibodies (see Table 1) were diluted 1:5000.

### 2.6. Western Blot

Cells were lysed in 50 µL ice-cold MOPS Triton buffer (Supplementary Table S2) and centrifuged at 13.000× *g* for 10 min. Afterward, 16.6% of total volume 6 × Laemmli buffer (Supplementary Table S2) was added and heated to 95 °C for 5–10 min. Proteins were separated by 12% sodium dodecyl sulfate–polyacrylamide gel electrophoresis (SDS-PAGE) (TNF, COX-2, vimentin) or 8% SDS-PAGE (collagen I, fibronectin, E-cadherin or N-cadherin) and transferred onto a nitrocellulose membrane. After blocking with 5% nonfat dry milk powder (A. Hartenstein GmbH, Würzburg, GER, CM35) in TRIS-buffered saline with Tween20 (TBS Tween20) (Supplementary Table S2), membranes were incubated with first antibody (Table 1) diluted in 5% bovine serum albumin (BSA) in TBS Tween20 overnight. Horseradish peroxidase (HRP)-coupled secondary antibodies (see Table 1), diluted 1:1000 in 5% nonfat dry milk powder in TBS Tween20, were used. After removal of the secondary antibody solution, three wash steps in TBS TWEEN20 were performed. Finally, Clarity™ Western ECL Substrate (Bio-Rad, Munich, Germany, #1705061) was added, and the peroxidase activity-based light emission was recorded by an imaging system (Image Quant LAS4000, GE Health care, Buckinghamshire, UK). Alternatively, IRDye-coupled fluorescent secondary antibodies (1:20,000 in 5% nonfat dry milk powder in TBS-Tween20; Li-Cor, Biosciences, Lincoln, NE, USA) were used and visualized with Odyssey infrared imaging system from Li-Cor, Biosciences. Density of protein bands was quantified using Quantity One software from BioRad (version 4.6.9, Bio-Rad, Munich, Germany).

### 2.7. Quantitative PCR

Isolation of ribonucleic acid (RNA) as well as reverse transcription of RNA were performed using a commercial kit from Invitrogen (Life Technologies, Darmstadt, Germany, 1062100300, B030S, M030L, 100000840, 1000425, Y02321) according to their instructions. Real-time PCR was performed using the SYBR Green reagent (Invitrogen by Thermo Fisher, Grand Island, NY, USA, 17733-046) and desoxynukleoside (VWR International GmbH, Darmstadt, Germany, 20-1011, 20-1021, 20-1031, 20-1041). Primers for PCR were synthesized by Microsynth AG (Balgach, Switzerland). Primer sequences and annealing temperatures are provided in Table 2. Fold change of gene expression was calculated by the 2^ΔΔCt^ method. β-actin expression was used as reference for the measurements with SYBR Green reagent.

Further methods such as determination of cytosolic pH, caspase-3 activity assay, transepithelial electric resistance and the MMP-activity assay are described in the Supplementary Material.

### 2.8. Data Analysis

All data are reported as mean ± s.e.m. Statistical significance was determined by unpaired Student’s *t* test or ANOVA, as appropriate. Differences were considered statistically significant when *p* < 0.05. The data analysis was performed with the software SigmaPlot (version 12.5, © Systat Software GmbH, Erkrath, Germany).

## 3. Results

If a change is significantly different compared to the control, the mean ± s.e.m. and the *p* value are given in brackets.

### 3.1. Extracellular Acidosis Reduces Cytosolic pH but Does Not Influence Tubule or Fibroblast Cell Viability

Cytosolic pH of NRK-52E cells were determined by using the pH-sensitive dye BCECF (2′,7′-bis-(2-carboxyethyl)-5-(and-6)-carboxyfluorescein, acetoxymethyl ester, (Invitrogen, Paisley, UK, B-3051)) as described before [28,29]. Under control conditions (pHe = 7.4), the intracellular pH was 7.28 ± 0.02 (*n* − (75)) and 6.61 ± 0.01; *p* < 0.001 (*n* − (75)) under acidic conditions (pHe = 6.0) (Supplementary Figure S1g). For NRK-49F cells, the respective values were 6.91 ± 0.01 (*n* − (50)) and 6.41 ± 0.02; *p* < 0.001 (*n* − (50)) (Supplementary Figure S1g). To exclude negative effects of acidic treatment on cell viability, cellular caspase-3 activity as an indicator for apoptosis was measured. Moreover, caspase-3 release through leaky plasma membranes into the media, as an indicator for necrosis, was analyzed. For the latter purpose, caspase-3 activity in the media was measured and expressed as a percentage of total caspase-3 activity (cellular + media activity). Under control conditions, caspase-3 activities were low but still reliably measurable. Thus, caspase-3 release can be used as an alternative biomarker for necrosis (instead of, e.g., LDH-release).

NRK-52E and NRK-49F cells were exposed to various pH values (7.4, 6.8, 6.6, 6.4, 6.2, and 6.0) over a period of 48 h. As shown in Supplementary Figures S1a–f and S2, acidosis did not induce major changes. Solely at pH 6.0, a slight decrease in caspase-3 release (33.3 ± 3.1 to 17.9 ± 2.7; *p* = 0.001) and a slight increase in caspase-3 activity (228 ± 59; *p* = 0.04) were observed in NRK-52E cells (Supplementary Figure S1c,e). Thus, cell viability was not reduced substantially by lower extracellular pH values.

### 3.2. Renal Epithelial Cells

#### 3.2.1. Markers of Cellular Differentiation

**Figure 1:**
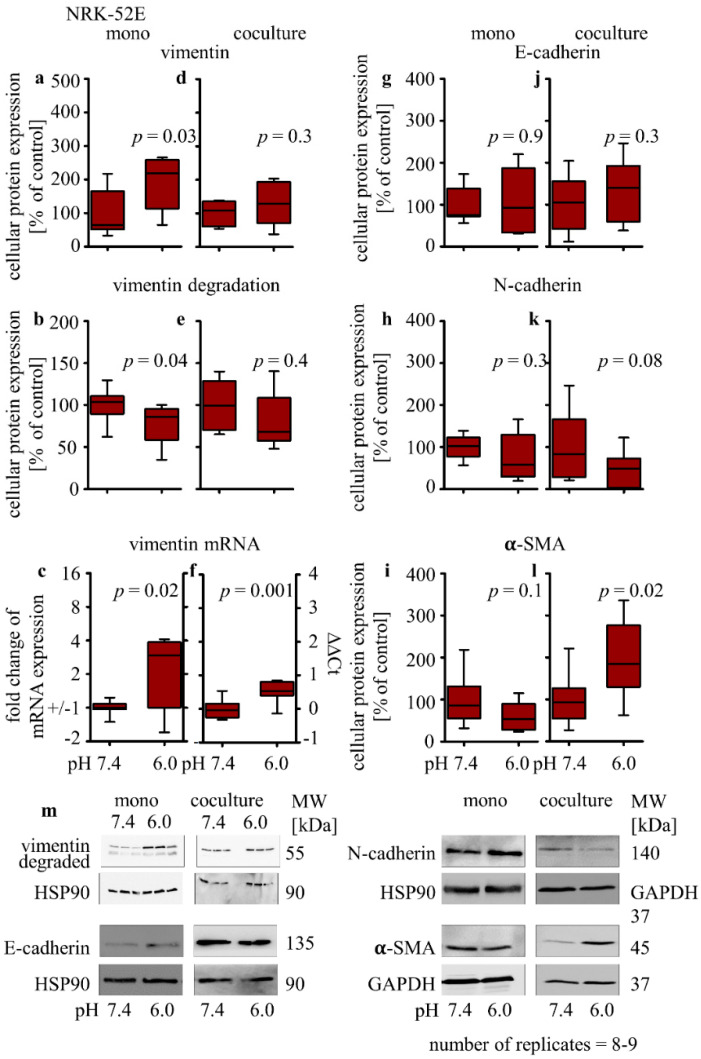
Acidosis effect on EMT markers in NRK-52E cells in mono- and co-cultures. Impact of acidosis on vimentin (**a**,**d**), E-cadherin (**g**,**j**), N-cadherin (**h**,**k**) and α-SMA (**i**,**l**) protein expression changes, vimentin degradation (**b**,**e**) and relative changes of vimentin mRNA expression (**c**,**f**). Representative Western blots of proteins isolated from cells exposed to acidosis (**m**). *n* − (8 − 9); exposure time = 48 h. *p* < 0.05 at significant difference compared with the control group.

First, the protein expression changes of vimentin, E-cadherin, N-cadherin, and α-SMA were analyzed by Western blot, as an indicator for EMT. Acidosis induced a significant increase in vimentin protein expression in monoculture (191% ± 27; *p* = 0.03, Figure 1a). Vimentin is subject to many posttranslational modifications such as phosphorylation and glycosylation. Furthermore, cysteinyl–aspartate-specific protease-mediated degradation of vimentin takes place during dedifferentiation and apoptosis, and it yields a specific fragment of ca. 48 kD besides the full-length vimentin of 57 kD [30]. Therefore, the lower band was used as a marker for vimentin degradation. In addition, degradation of the vimentin protein was reduced (77% ± 7.6; 0.04, Figure 1b), while the expression of the vimentin-coding mRNA was increased (1 ± 0.4; *p* = 0.02) (Figure 1c). No significant changes of E-cadherin, N-cadherin or α-SMA protein expression were observed (Figure 1g–i). Under co-culture conditions, vimentin protein expression and degradation were not affected by acidosis (Figure 1d,e), although the expression of vimentin-coding mRNA was still increased (0.5 ± 0.1; *p* = 0.001, Figure 1f). E-cadherin protein expression was also not affected, whereas N-cadherin protein expression was slightly decreased (46 ± 14; *p* = 0.08), and the expression of α-SMA protein was significantly increased (194 ± 31; *p* = 0.02) (Figure 1j–l).

#### 3.2.2. Distribution of E-Cadherin and Cellular Morphology

**Figure 2:**
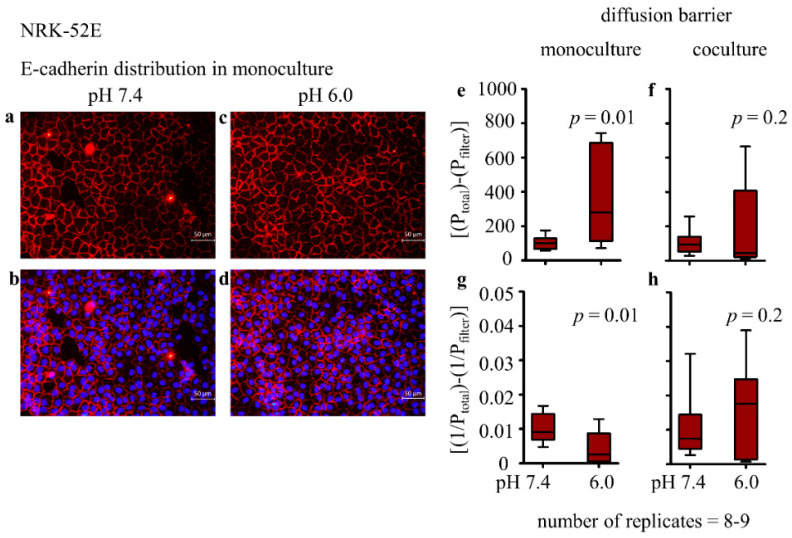
Impact of acidosis on E-cadherin distribution in NRK-52E in monoculture. (**a**–**d**) *n* − (24). Acidosis effect on barrier function of NRK-52E cells in mono-and co-culture (**e**–**h**). *n* − (9); exposure time = 48 h. *p* < 0.05 at significant difference compared with the control group.

E-cadherin is the main component of adherens junctions. Therefore, it can influence the cellular diffusion barrier. Although E-cadherin protein expression was not altered, its distribution can be disturbed and can thus reduce the diffusion barrier function. Therefore, we determined the distribution of E-cadherin by immunofluorescence. Under control conditions, 12.3% ± 1.2 of the total E-cadherin protein was localized in the cytosol (Figure 2a,b, Table 3).

After incubation with acidic media, 25.5% ± 1.6; *p* = 0.001 of the total E-cadherin was localized in the cytosol (Figure 2c,d and Table 3). This result shows that acidosis induced a certain redistribution of E-cadherin toward the cell interior. Moreover, the cell shape is crucial for the barrier function and depends among others for E-cadherin distribution. A measure for cell shape is the structure index (SI), or circularity. The SI was calculated by the formula SI = 4 πA/*p*^2^. Values close to 0.85 indicate square- or cobblestone-like-shaped cells typical for epithelial cells, and values near 0.4 indicate spindle-shaped cells typical for fibroblasts or smooth muscle cells [31]. The present data show that NRK-52E cells under control conditions display an SI of 0.86, corresponding to their epithelial character (Table 3). Conversely, the SI decreased to 0.75 ± 0.02; *p* < 0.001 after incubation with acidic media. This indicates a slight morphological change that can influence the cellular barrier function (Table 3). It is also described that during the development of EMT, the apparent membrane thickness can increase. This was also shown in the presented study (Table 3).

#### 3.2.3. Epithelial Permeability and Transepithelial Electrical Resistance (TEER)

As a result of a progressed EMT, the epithelial barrier function can be reduced. To assess this function further, we determined the permeability of the cell monolayer using 70 kDa FITC-dextran. As shown in Figure 2e, permeability for dextran was significantly higher under acidic conditions, confirming a reduction of epithelial tightness. The slope of the diffusion curves *p* was 101 ± 12 (fluorescence units/(L/h); *n* − (9)) under control conditions, 366 ± 85; *p* = 0.01 under acidic conditions (*n* − (9)) and 1047 ± 123 for filters without cells (*n* − (6)). Calculation of the barrier function ((1/Ptotal)-(1/Pfilter)) shows that the barrier function dropped from 0.010 ± 0.001 at pH 7.4 to 0.004 ± 0.001, *p* = 0.01 at pH 6.0 (2g). As shown in Figure 2f, permeability for dextran was unchanged under acidic conditions in co-culture, confirming the stability of epithelial tightness. In addition, the calculated barrier function was not affected by acidosis in co-culture (Figure 2h).

Transepithelial electric resistance (TEER) of NRK-52E cells under control conditions (pH 7.4, serum-free media for 48 h) was 128 ± 34 Ω cm^2^ (*n* − (9)), a value typical for a low-resistance epithelium such as the proximal tubule. Under acidic conditions, TEER was slightly lower compared to control conditions (102 ± 17 Ω cm^2^; *p* = 0.06) (Supplementary Figure S3a). Acidosis did not affect TEER of NRK-52E cells under co-culture conditions (Supplementary Figure S3b).

#### 3.2.4. Influence of Extracellular Acidosis on Inflammation Marker

**Figure 3:**
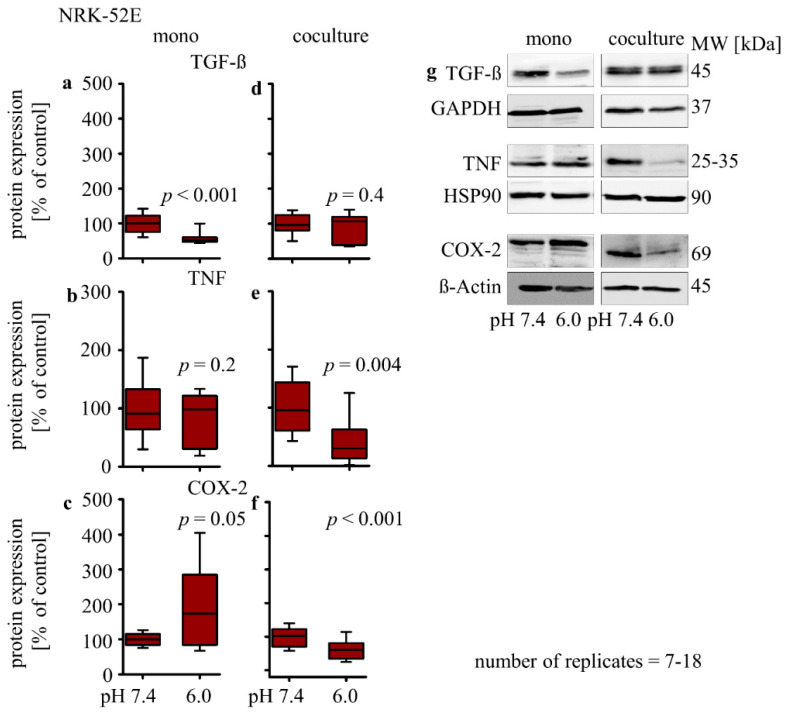
Acidosis effect on inflammation markers in NRK-52E cells in mono-and co-culture. Impact of acidosis on TGF-β (**a**,**d**), TNF (**b**,**e**), and COX-2 (**c**,**f**); representative Western blots of proteins isolated from cells exposed to acidosis (**g**). *p* < 0.05 at significant difference compared with the control group; *n* − (7 − 18). Exposure time = 48 h.

The cytokines TGF-β and TNF as well as COX-2 metabolites are often involved in nephropathic processes, acting as intercellular mediators. To assess whether their expression might be affected by extracellular acidosis, we determined protein expression changes of TGF-β, TNF and COX-2. In monoculture, the acidic media led to a decrease in TGF-β protein expression (58% ± 7.2; *p* < 0.001), had no impact on the expression of the TNF protein, and led to an increase in COX-2 protein expression (192% ± 41; *p* = 0.05) (Figure 3a–c). In co-culture, the acidic media had no impact on the TGF-β protein expression and led to a decreased expression of the TNF (40% ± 12; *p* < 0.001) and COX-2 (62% ± 7; *p* = 0.004) protein (Figure 3d–f).

#### 3.2.5. Collagen I and Fibronectin Expression

**Figure 4:**
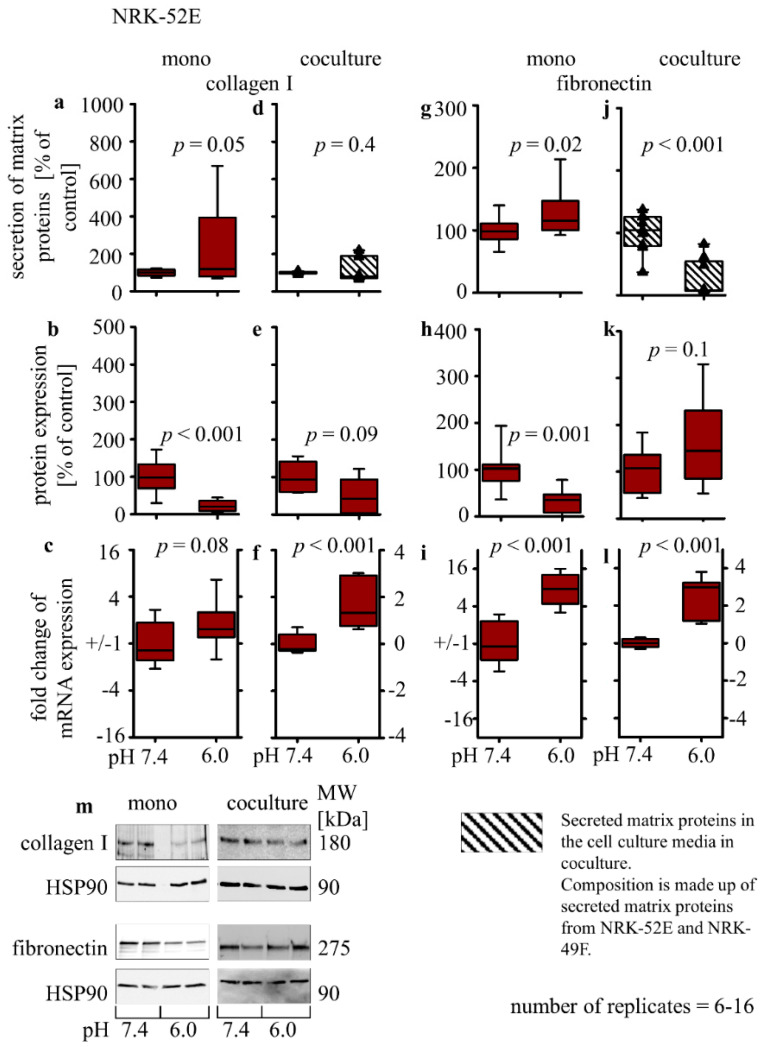
Acidosis effect on fibrosis markers in NRK-52E cells in mono- and co-cultures. Impact of acidosis on the expression of secreted collagen I (**a**,**d**) and fibronectin (**g**,**j**). Intracellular protein expression changes of collagen I (**b**,**e**) and fibronectin (**h**,**k**) as well as relative changes of collagen I (**c**,**f**) and fibronectin (**i**,**l**) mRNA expression. (**m**) Representative Western blots of proteins isolated from cells exposed to acidosis. *p* < 0.05 at significant difference compared with the control group; *n* − (6 − 16). Exposure time = 48 h.

Forty-eight hour exposure of NRK-52E cells to acidic media (pH 6.0) resulted in a slightly increased secretion of collagen I (234% ± 62; *p* = 0.05) and fibronectin (130% ± 11; *p* = 0.02) (Figure 4a,g). In contrast, Western blot analysis showed a significantly reduced expression of intracellular collagen I (22% ± 5; *p* < 0.001) and fibronectin (34% ± 8; *p* < 0.001) after exposure to pH 6.0 (Figure 4b,h). Finally, the expression of collagen I (0.8 ± 0.3; *p* = 0.08) and fibronectin (2.9 ± 0.3; *p* < 0.01)-coding mRNAs was increased in monoculture, after incubation with acidic media (Figure 4c,i).

The effect of acidosis on secreted collagen I and fibronectin in co-culture cannot be assessed for the two cell types separately. Therefore, the assessment of the contribution of only an individual cell type is not possible. Under co-culture conditions, exposure to acidic media had no impact on total extracellular collagen I but led to a decrease in total extracellular fibronectin (26% ± 10; *p* < 0.01) (Figure 4d,j). The intracellular protein amount of collagen I or fibronectin was not altered (Figure 4e,k), whereas the expression of collagen I (1.6 ± 0.3; *p* < 0.001) and fibronectin-coding mRNA (2.5 ± 0.4; *p* < 0.001) was increased (Figure 4f,l).

### 3.3. Renal Fibroblast

#### 3.3.1. Markers of Cellular Differentiation

**Figure 5:**
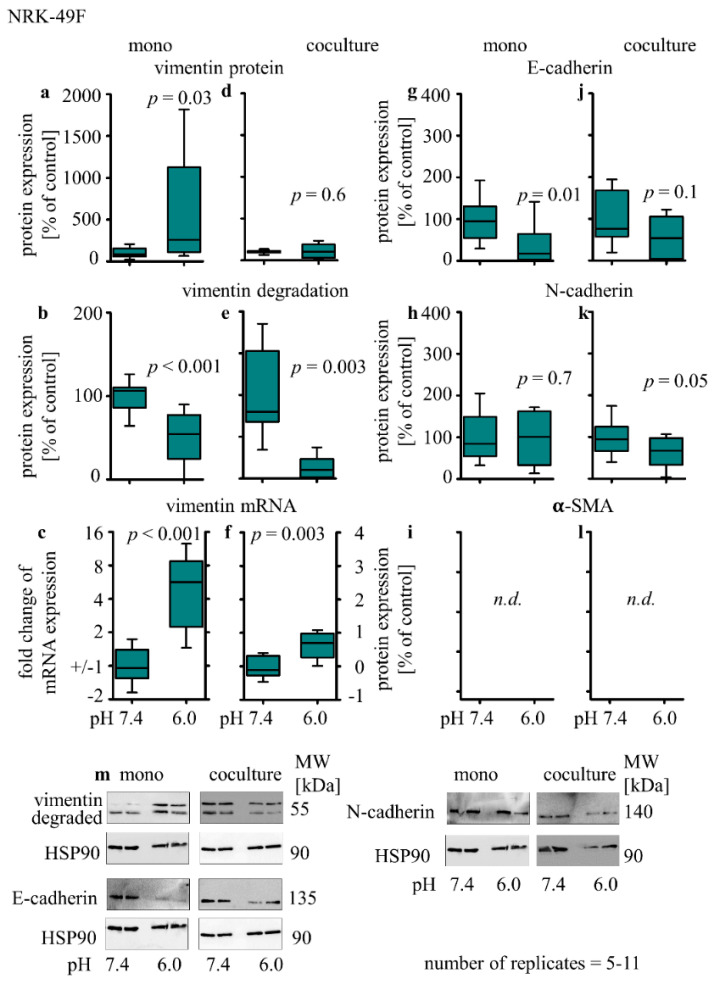
Acidosis effect on EMT markers in NRK-49F cells in mono- and co-cultures. Impact of acidosis on vimentin (**a**,**d**), E-cadherin (**g**,**j**), N-cadherin (**h**,**k**) and α-SMA (**i**,**l**) protein expression changes, vimentin degradation (**b**,**e**) and relative changes of vimentin mRNA expression (**c**,**f**). Representative Western blots of proteins isolated from cells exposed to acidosis. (**m**) *p* < 0.05 at significant difference compared with the control group; *n* − (5 − 11). Exposure time = 48 h.

In monoculture, acidosis induced an increase in vimentin protein expression (541% ± 238; *p* = 0.03 Figure 5a), a decrease in vimentin degradation (57% ± 9; *p* = 0.001) and an increase in vimentin mRNA expression (2.3 ± 0.4; *p* < 0.001). Furthermore, a decrease in E-cadherin protein expression (39% ± 15; *p* = 0.01 Figure 5g) and no change of N-cadherin protein expression was measured (Figure 5h). α-SMA protein expression could not be detected reliably in NRK-49F with the used antibody (Figure 5i). Under co-culture conditions, acidosis had no impact on vimentin protein expression (Figure 5d), whereas vimentin protein degradation was reduced (13% ± 6; *p* = 0.003), and the expression of vimentin-coding mRNA was increased (0.6 ± 0.1; *p* = 0.003) (Figure 5e–f). Moreover, in co-culture, an acidosis-induced decrease in N-cadherin (63% ± 11; *p* = 0.05, Figure 5k) protein expression was observed, whereas the E-cadherin protein expression was unchanged (Figure 5j).

#### 3.3.2. Influence of Extracellular Acidosis on Inflammation Marker

**Figure 6:**
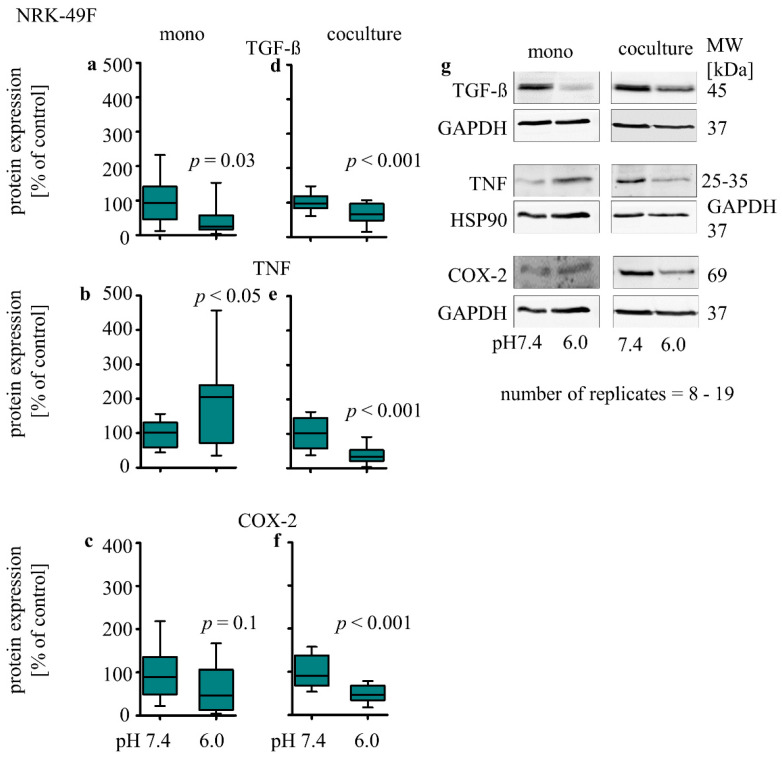
Acidosis effect on inflammation markers in NRK-49F cells in mono- and co-cultures. Impact of acidosis on TGF-β (**a**,**d**), TNF (**b**,**e**), COX-2 (**c**,**f**). Representative Western blots of proteins isolated from cells exposed to acidic media (**g**). *p* < 0.05 at significant difference compared with the control group; *n* − (8 − 19). exposure time = 48 h.

In monoculture, the expression of TGF-β protein (39% ± 15; *p* = 0.03, Figure 6a) was decreased, TNF protein (204% ± 45; *p* = 0.05, Figure 6b) expression was increased, and COX-2 protein expression was unchanged (Figure 6c). In co-culture, acidosis caused a decrease in TGF-β (59% ± 8; *p* < 0.01, Figure 6d), TNF (36% ± 7; *p* < 0.01, Figure 6e) and COX-2 protein (50% ± 5; *p* < 0.001, Figure 6f) expression.

#### 3.3.3. Collagen I and Fibronectin Expression

**Figure 7:**
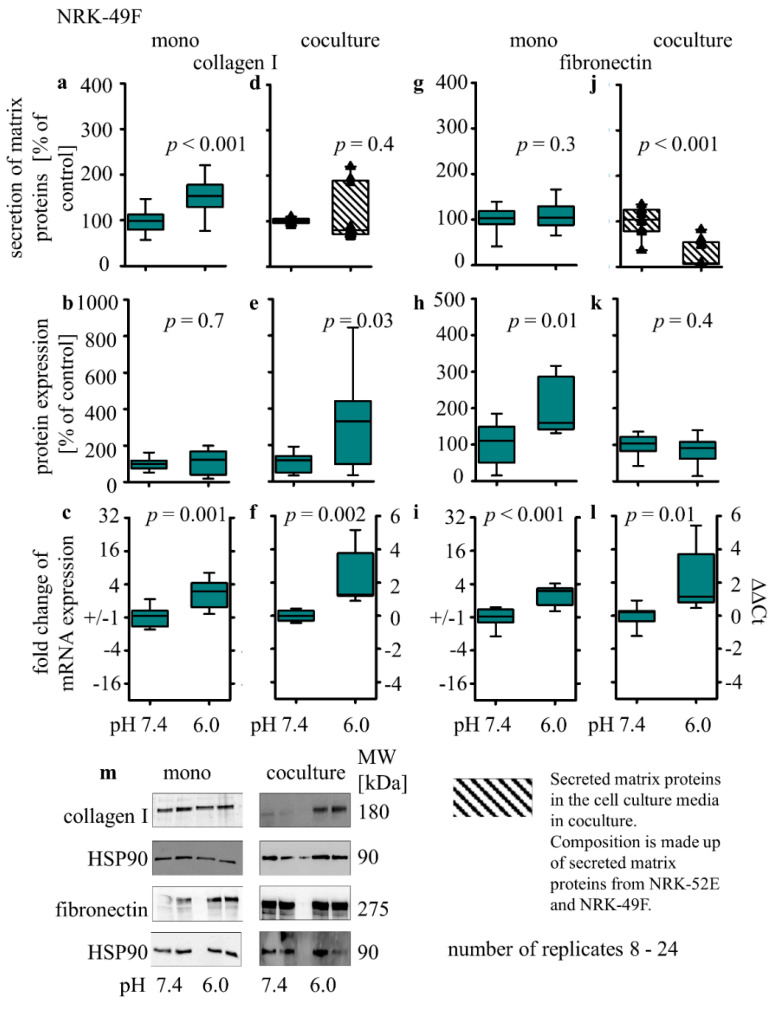
Acidosis effect on fibrosis markers in NRK-49F cells in mono- and co-cultures. Impact of acidosis on the expression of extracellular collagen I (**a**,**d**) and fibronectin (**g**,**j**). Intracellular protein expression changes of collagen I (**b**,**e**) and fibronectin (**h**,**k**) as well as relative changes of collagen I (**c**,**f**) and fibronectin (**i**,**l**) mRNA expression. Representative Western blots of proteins isolated from cells exposed to acidosis (**m**). *p* < 0.05 at significant difference compared with the control group; *n* − (8 − 24). Exposure time = 48 h.

In NRK-49F cells, the acidic media led to an increase in collagen I secretion (153% ± 12; *p* < 0.001, Figure 7a) but had no impact on the secretion of fibronectin (Figure 7g). Intracellular collagen I protein was unchanged (Figure 7b), but the intracellular fibronectin expression was increased (208 ± 30; *p* = 0.01, Figure 7h). The results show that acidic media led to an increase in the expression of collagen I- (mono 1.5 ± 0.3; *p* < 0.001; co 2.1 ± 0.6; *p* < 0.002, Figure 7c,f) or fibronectin- (mono 1.3 ± 0.3; *p* < 0.001; co 2.0 ± 0.7; *p* = 0.01, Figure 7i,l) coding mRNA under mono- and co-culture conditions. Incubation with acidic media in co-culture causes an increase in intracellular collagen I (313% ± 86; *p* = 0.03, Figure 7e) and no impact on fibronectin protein expression (Figure 7k).

## 4. Discussion

According to the German Society of Nephrology, 8–12% of all chronic kidney diseases (CKD) are the result of tubule–interstitial nephropathies [32]. These comprise a variety of clinical entities, e.g., tubule–interstitial nephritis, diabetic nephropathy, proteinuria-induced interstitial damage, obstructive uropathy or balkan nephropathy [32]. For these entities, disease-pattern inflammation, EMT and fibrosis are frequent. Inflammation and fibrosis are accompanied by changes of the micromilieu, including extracellular acidosis [17,18,19]. Subsequently, the acidic alteration of the micromilieu will influence the surrounding tubule as well as the interstitial cells, thereby initiating a self-enhancing vicious cycle and accelerating the decline of renal function.

Besides that, it is well established that the crosstalk between the proximal tubule and fibroblasts (tubule–interstitial crosstalk) is of major relevance for physiological and pathophysiological processes, e.g., during formation of cell junctions or matrix protein homeostasis and thus during the development of inflammation, EMT and fibrosis. Communication between proximal tubule epithelial cells and fibroblasts, mediated by soluble mediators such as growth factors and cytokines, is crucial for the homoeostasis of tubular cell function, whereas changes of this crosstalk can support the development of tubular–interstitial diseases [33,34].

In the present study, we investigated the potential impact of tubule–interstitial acidosis, represented by an acidic media pH, on the development of inflammation, EMT and fibrosis in epithelial cells derived from the proximal tubule (NRK-52E) and renal fibroblasts (NRK-49F) in mono- and co-cultures. A former study provided evidence that NRK-52E and NRK-49F cells develop more physiological phenotypes in co-culture, showing the advantage and even necessity of using a co-culture model [35].

The EMT of proximal tubule cells and activation of fibroblasts are involved in the development of CKD. A hallmark of activated fibroblasts is the increased expression of mesenchymal proteins [36,37]. The present study shows that under monoculture conditions, the expression of vimentin protein was drastically increased in NRK-52E as well as NRK-49F cells. This is comparable to the acidosis impact on other cell types such as melanoma or distal tubule cells [38,39]. Moreover, acidosis led, in both cell types, to an increase in the expression of vimentin-coding mRNA, indicating an underlying transcriptional mechanism. Besides, acidosis led to a decrease in vimentin degradation in both cell lines. Vimentin degradation is orchestrated by the interaction of enzymes such as kinases, phosphatases or transferases [40,41] known to be modulated by extracellular acidosis [42,43]. The smaller fragment is not only a degradation product, but it seems to exert an intracellular signaling function of its own. This has been shown before by other studies [44,45]. In contrast, under co-culture conditions, the acidosis-induced increase in vimentin protein expression was significantly diminished in NRK-52E and NRK-49F cells. These data show that the acidosis-induced impact on vimentin protein expression depends on cellular crosstalk. Surprisingly, this was not the case for the acidosis-induced impact on vimentin-coding mRNA, indicating a certain specificity of the crosstalk. In the case of vimentin, it acts more on posttranscriptional events.

The expression of cadherins and α-SMA in NRK-52E cells in monoculture was not affected by acidosis. However, we observed an impact on the subcellular distribution of E-cadherin, indicative of disturbed adherens junctions and therefore reduced epithelial tightness, typical for EMT. Moreover, we measured a change in the cell shape from square-shaped cells to more spindle-like-shaped cells. Besides that, the membrane thickness was increased, which is also typical for EMT. In contrast, under co-culture conditions, acidosis led to a slight decrease in the epithelial marker protein N-cadherin and an increase in the mesenchymal marker protein α-SMA. These results show that extracellular acidosis modulates the development of partial EMT in a complex manner dependent on cellular crosstalk. Our findings are consistent with former studies showing that the development of EMT depends on cellular crosstalk [35].

Acidosis reduced the expression of the E-cadherin protein but not of N-cadherin in fibroblasts in monoculture. Under co-culture conditions, acidosis still reduced E-cadherin expression and induced a decrease in N-cadherin. It is known that cadherins have an important functional role in activated fibroblasts, as they participate in cell–cell adhesion, necessary for communication with surrounding cells, invasion and migration [46,47]. Thus, the observed changes indicate that fibroblasts are quieter during acidosis in co-culture. It was also shown by Lovisa et al. that EMT can be accompanied by reduced expression of certain transporters, namely Na^+^/K^+^-ATPase and organic anion transporters [9]. Therefore, we analyzed mRNA expression of the essential Na^+^/K^+^-ATPase β subunit (ATP1B1) and of the organic anion transporter 3 (OAT3) under acidotic conditions but did not observe significant changes (Supplementary Figure S5a, see supplementary Table S1 for primer sequences).

Activated fibroblasts can lead to altered secretion of inflammatory mediators [48]. Thus, we measured whether acidosis has an impact on the expression of inflammatory marker proteins. In monoculture, acidosis provoked an inflammatory response of both cell lines. In favor of this, an increased protein expression of the inflammatory enzyme COX-2 and a decrease in the anti-inflammatory cytokine TGF-β was measured in NRK-52E, with an increase in TNF protein expression in NRK-49F cells. Furthermore, the expression of COX-2 mRNA was increased after exposure to acidic media (Supplementary Figure S5a, see supplementary Table S1 for primer sequences). These results suggest that extracellular acidosis mediates the inflammatory response in part by transcriptional mechanisms [49,50,51]. Under co-culture conditions, the acidosis-induced inflammatory response was absent, and the expression of the inflammatory marker proteins was even decreased. These results suggest that the crosstalk between tubule and fibroblast cells has a protective impact regarding an inflammatory response.

With regard to fibrosis, acidosis led to an increase in secreted collagen I in both cell lines in monoculture and of secreted fibronectin in NRK-52E cells in monoculture. In contrast, acidosis had no impact on the amount of extracellular collagen I, and even led to a decrease in extracellular fibronectin under co-culture conditions. However, it is not possible to assign these changes in the media reliably to one of the two cell types. These data suggest that cellular crosstalk protects against the development of a fibrotic milieu. Moreover, our aim was to unveil possible mechanisms leading to the fibrotic response in monoculture. For NRK-52E cells, we showed that more collagen I and fibronectin was secreted and mRNA expression was increased, indicating a transcriptional regulation. This is also the case for collagen I in fibroblasts. In contrast, intracellular collagen I and fibronectin in NRK-52E cells were decreased.

These changes can be a result of posttranscriptional regulation. Possible mediators of posttranscriptional regulations are microRNAs. Other possible explanations for the observed changes are altered collagen I and fibronectin secretion or degradation rates. The progression of fibrosis can also be supported by altered activity of matrix metalloproteinases (MMP) [52,53]. The data of the present study show that in NRK-52E cells, MMP activity was slightly decreased under co-culture conditions, compared to the situation in monoculture (Figure S4). Additionally the incubation of NRK-52E cells in co-culture with acidic media led to a further decrease in MMP activity (Figure S4a,e). Thus, changed MMP activity cannot explain the observed changes of matrix proteins and seems not to play a major role for the acidosis-induced effects observed here. We cannot exclude that individual members of the MMP family are affected, as we measured only the overall activity of MMPs.

In summary, we showed that intercellular crosstalk between epithelial and fibroblast cells has a protective function regarding the development of acidosis-induced inflammatory and fibrotic alterations in proximal tubule cells and renal fibroblasts. Therefore, it is of major importance to apply the appropriate cell culture system to evaluate pathophysiological mechanisms. Under these conditions, we could show that acidosis has a complex impact on epithelial cells and fibroblasts, which requires further investigation. Of special relevance is a putative modulatory effect of acidosis on other pathological stimuli. Furthermore, impaired crosstalk in vivo can reduce tissue resilience and convert innocuous stimuli into pathogenic drivers. Thus, it will be the crucial aim of advanced studies to identify underlying mechanisms. Intracellular signaling can be affected, and further studies can show that acidosis acts through an MAPK-dependent mechanism [54]. Conversely, the question remains of how the cellular crosstalk is executed. This study showed that the protein amount of COX-2 was changed. Moreover, the COX-2 metabolites represent soluble mediators. Thus, COX-2 metabolites are promising candidates, which should be investigated in future studies. Besides these directed approaches, it can be insightful to follow an indirect approach. mRNA sequencing analyses can reveal acidosis and co-culture-dependent genes. Conversely, the conditioned media from co-culture can be analyzed with mass spectroscopy to unmask soluble mediators that execute the cellular crosstalk.

It should be kept in mind that this study was performed with rat cell lines. The kidneys of rats differ from human kidneys. Therefore, the next step to approximate the in vivo situation should be to create a proper human co-culture system.

## Figures and Tables

**Table 1:** Antibodies, order number, host and dilutions used.

Target	Company	Order Number	Host	Dilution
anti-mouse IRDye 800CW	Li-cor Biosciences GmbH, Bad Homberg, Germany	926-32210	Goat	1:40,000
anti-mouse Alexa Fluor^®^ 594	Invitrogen by Thermo Fisher, Grand Island, NY, USA)	A21203	donkey	1:1000
Anti-Rabbit IgG HRP	Cell Signaling, Danvers, MA, USA	7074	Goat	1:1000
β-actin	Cell Signaling, Danvers, MA, USA	3700	Mouse	1:1000
Collagen I	Biozol, Eching, Germany	PAB 17205	Rabbit	1:1000
COX-2	Abcam, Cambridge, UK	ab52237	Rabbit monocl	1:500
E-Cadherin	Cell Signaling, Danvers, MA, USA	14472	Mouse	1:500
Fibronectin	Biomol, Hamburg, Germany	600-401-117-0.1	Rabbit	1:1000
GAPDH	Cell Signaling, Danvers, MA, USA	2118	Rabbit	1:1000
HSP90	Cell Signaling, Danvers, MA, USA	4874	Rabbit	1:1000
N-cadherin	Cell Signaling, Danvers, MA, USA	14215	Mouse	1:500
α-SMA	Cell Signaling, Danvers, MA, USA	14968	Rabbit	1:500
TGF-β	Cell Signaling, Danvers, MA, USA	3711	Rabbit	1:500
TNF-α	Cell Signaling, Danvers, MA, USA	6945	Rabbit	1:200
Vimentin	Cell Signaling, Danvers, MA, USA	5741	Rabbit	1:1000

Abbreviations: COX-2, prostaglandin-endoperoxide synthase 2/cyclooxygenase 2; GAPDH, glyceraldehyde 3-phosphate dehydrogenase; HRP, horseradish peroxidase; HSP90, heat shock protein 90; TGF-β, transforming growth factor-β; TNF-α, tumor necrosis factor α.

**Table 2:** Primer sequences and annealing temperatures.

Gene Name	Accession Number	Forward 5′-3′	Backward 5′-3′	Annealing Temperature °C
Actb	NM_031144.2	TGACGGTCAGGTCATCACTATC	GGCATAGAGGTCTTTACGGATG	56 °C
Col1a1	NM_053304.1	CGGCTCCTGCTCCTCTTAG	GCCATTGTGGCAGATACAGA	58 °C
Fn1	NM_019143	CACCGAAACCGGGAAGAG	TTGCCTAGGTAGGTCCGTTC	58 °C
Vim	NM_031140.1	AATGACCGCTTCGCCAACTA	GGTCAAGACGTGCCAGAGAA	60 °C

Abbreviations: Actb, β-Actin; Col1a1, collagen type I; Fn1, fibronectin; Vim, vimentin.

**Table 3:** Cytosolic E-cadherin and cell morphology.

	pH 7.4 (Mean ± s.e.m.)	pH 6.0 (Mean ± s.e.m.)	*p* Value
Cytosolic E-cadherin (%)	12.3 ± 1.2	25.5 ± 1.6	0.0000001
Structure index (SI)	0.86 ± 0.01	0.75 ± 0.03	0.0000819
Membrane thickness (AU)	49 ± 0.01	71 ± 0.02	0.0000003

## Data Availability

The datasets used and/or analyzed during the current study are available from the corresponding author on reasonable request.

## Supplementary Materials

The following supporting information can be downloaded at: https://www.mdpi.com/article/10.3390/biomedicines10030681/s1, Figure S1: Impact of acidosis on cytosolic pH and viability; Figure S2: Impact of acidosis on protein amount in co-culture; Figure S3: Impact of acidosis on transepithelial electrical resistance (TEER); Figure S4: Impact of acidosis on MMP-activity; Figure S5: Impact of acidosis on mRNA expression; Table S1 Primer sequences and annealing temperatures; Table S2 buffer composition.
