# Supplementary material for: Epithelial–Fibroblast Crosstalk Protects against Acidosis-Induced Inflammatory and Fibrotic Alterations
Source: Biomedicines. 2022 Mar 16;10(3):681. doi: 10.3390/biomedicines10030681 (PMC8945333; doi:10.3390/biomedicines10030681)
Supplement: Supplementary file 1 [file biomedicines-10-00681-s001.zip › biomedicines-1624562-supplementary.pdf]

Supplementary material table of content

Supplementary figure S1 (S1): Impact of acidosis on cytosolic pH and viability

Supplementary figure S2 (S2): Impact of acidosis on protein amount in coculture

Supplementary figure S3 (S3): Impact of acidosis on transepithelial electrical resistance (TEER)

Supplementary figure S4 (S4): Impact of acidosis on MMP-activity

Supplementary figure S5 (S5): Impact of acidosis on mRNA expression

Supplementary Table S1 Primer sequences and annealing temperatures.

Supplementary table S2 buffer composition

S1

Determination of cytosolic pH.

Cytosolic pH (pH_i) of single cells was determined using the pH-sensitive dye BCECF (2',7'-bis-(2-carboxyethyl)-5-(and-6)-carboxyfluorescein, acetoxymethyl ester, (Invitrogen, Paisley, UK, B-3051) as described before [1, 2] . In brief, cells were incubated with Ringer solution (Supplementary table S2) containing 5 μM BCECF-AM for 15 min. Afterwards, the cover slips were rinsed 2 times with superfusion solution to remove the excess of the dye and transferred to the stage of an inverted Axiovert 100 TV microscope (Zeiss, Oberkochen, GER). Excitation light source was a 100 W mercury lamp. The excitation wavelengths were 450 nm / 490 nm, the emitted light was filtered through a bandpass-filter (515-565 nm). The data acquisition rate was one fluorescence intensity ratio every 10 s or 5 s, respectively. After background subtraction, fluorescence intensity ratios were calculated. pH calibration was performed after each experiment by the nigericin (Sigma-Aldrich, Munich, GER, N7143) technique [3, 4] using a two-point calibration (pH 6.8 and 7.5). The calibration solutions contained 132 mM KCl (Merck KGaA, Darmstadt, GER , 04936) and 1 mM $CaCl_2$ (Merck KGaA, Darmstadt, GER, 1.02382.1000), 1 mM $MgCl_2$ (Sigma-

Aldrich, Munich, GER, M-9272) , 10 mM HEPES (Sigma-Aldrich, Munich, GER, H3034) and 10 µM nigericin.

Caspase-3 activity

To draw conclusions about apoptosis the caspase-3-activity was measured. First cells were lysed in 100 µl 3-(N-morpholino)propanesulfonic acid (MOPS)-triton buffer (Supplementary table S2) and afterwards centrifuged for 10 min at 13.000 g and 4 °C. The samples were separated in cell pellet and supernatant, containing the enzyme. 60 µl of the supernatant were incubated with the caspase-3 substrate DEVD-7-amino-4 trifluoromethylcoumarin (DEVD-AFC) (Biomol GmbH, Ger, ABD-13401), at 37 °C, for 90 min. In the reaction buffer the end concentration of DEVD-AFC was 40 µM Afterwards the fluorescence of the cleaved product, AFC, was measured after 30, 60 and 90 min at 400 nm excitation and 505 nm emission wavelength in a multiwall reader (Infinite® M200; Tecan 2.5 Männedorf, Switzerland). AFC was quantified by a calibration curve using known AFC concentrations. Protein content was determined with bicinchoninic acid assay [5] from Pierce (Thermo Fisher scientific, GER). To obtain information about necrosis the release of caspase-3 was determined. Therefore, the caspase-3 activity was measured in the cell culture media. To get an impression of the caspase-3 release, a ratio between caspase-3 activity obtained in media and total caspase-3 activity obtained in media and cell lysate was calculated.

Extracellular acidosis reduces cytosolic pH but does not influence cell viability

Cytosolic pH of NRK-52E cells determined by using the pH-sensitive dye BCECF (2',7'-bis-(2-carboxyethyl)-5-(and-6)-carboxyfluorescein, acetoxymethyl ester, Invitrogen, Paisley, UK) as described before [1, 2]. Under control conditions (pHe-(7.4)) the intracellular pH was 7.28 ± 0.02 (n-(75)) and 6.61 ± 0.01; $p < 0.001$ (n-(75)) under acidic conditions (pHe = 6.0) (S1g). For NRK-49F cells, the respective values were 6.91 ± 0.01 (n-(50)) and 6.41 ± 0.02; $p < 0.001$ (n-(50)) (fig. S 1g). To exclude negative effects of acidic treatment on cell viability, cellular caspase-3 activity as indicator for apoptosis was measured. Moreover, caspase-3 release through leaky plasma membranes, into the media as indicator for necrosis was analyzed. For the latter purpose caspase-3 activity in the media was measured and expressed as percentage of total caspase-3 activity (cellular + media activity). Under control conditions, caspase-3 activities were

low but still reliably measurable. Thus, caspase-3 release can be used as an alternative biomarker for necrosis (instead of e.g. LDH-release).

NRK-52E and NRK-49F cells were exposed to various pH values (7.4; 6.8; 6.6; 6.4; 6.2; 6.0) over a period of 48 h. As shown in fig. S 1a-f, acidosis did not induce major changes. Solely at pH 6.0 a slight decrease of caspase-3 release (33.3±3.1 to 17.9±2.7; p=0.01) and a slight increase in caspase-3 activity (228±59; p=0.04) was observed in NRK-52E cells (fig. S 1c, e). Thus, cell viability was not reduced substantially by lower extracellular pH values.

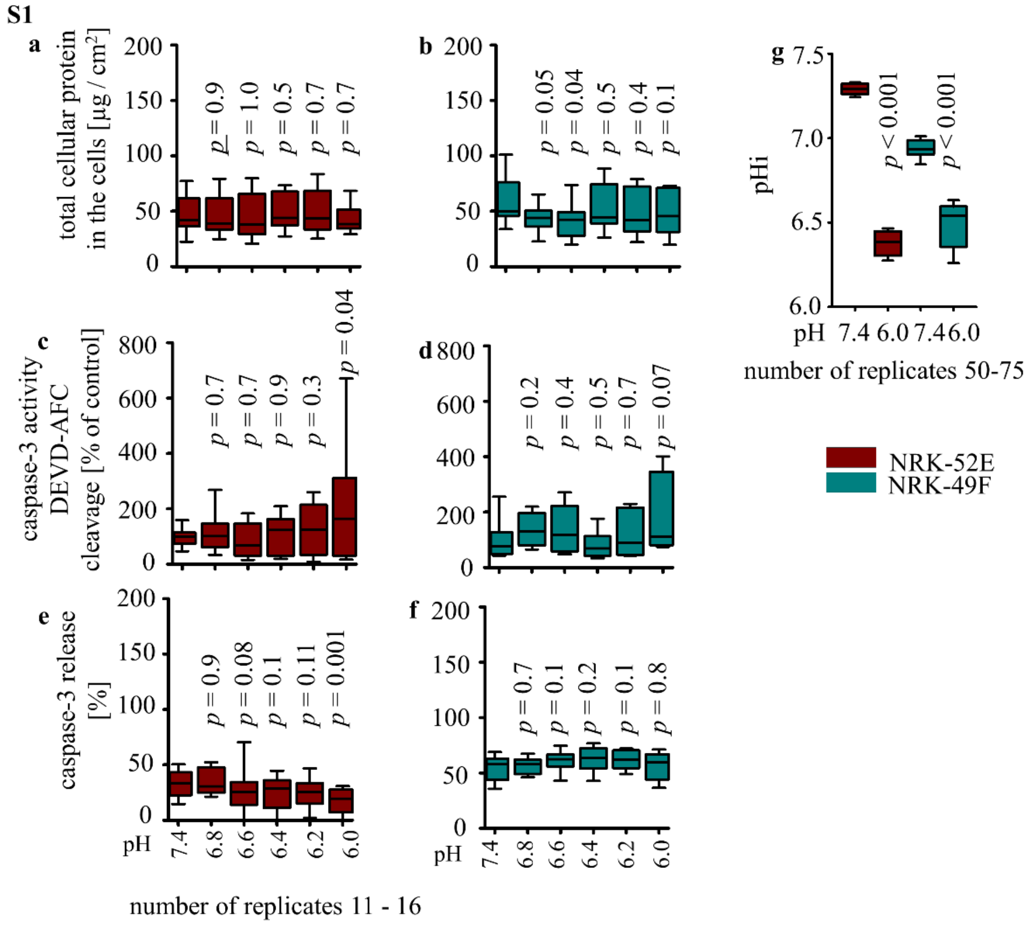

Supplementary figure S1 (S1): Impact of acidosis on viability and cytosolic pH in monoculture. Effect of acidosis on BCA (a, b), caspase-3 activity (c, d), caspase-3 release (e, f), and intracellular pH-value (g). p<0.05 significant difference compared with the control group, n-(8-9). exposure time=48 h.

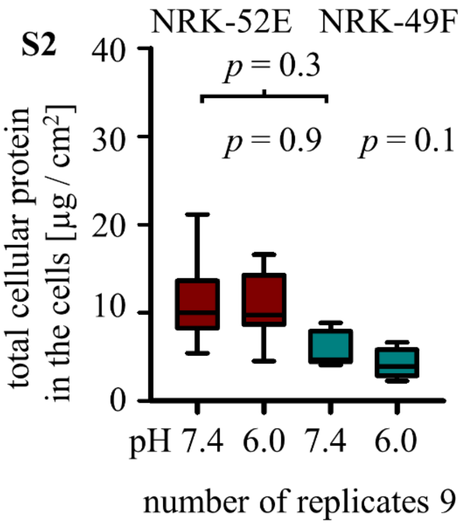

Supplementary figure S2 (S2): Impact of acidosis on total protein amount of NRK-52E and NRK-49F in coculture. $p < 0.05$ significant difference compared with the control group, n-(8-9). exposure time = 48 h.

Figure S2 shows that acidosis had no influence on the total protein amount in NRK-52E and NRK-49F cells. Moreover, this figure shows that NRK-52E cells, which grew on filter inserts, had a comparable total protein amount (47 µg/well ± 6) as NRK-49F cells (55 µg/well ± 6) which grew on the bottom of the plates.

S3 Transepithelial electric resistance (TEER)

TEER was measured using an epithelial voltohmmeter (EVOM) (world precision instruments, GER). The epithelial cells were cultivated as described in "2.2 experimental setup". The electrodes of the EVOM were placed at one hand into the apical medium site (above the filter) and on the other hand into the basolateral site (below the filter). TEER was measured before incubation with acidic media, after 24 h and again after 48 h incubation time. As background control, the TEER value of a well without cells was measured and subtracted from the other values. Results are shown as relative difference from the baseline TEER values at t = 0, which was considered as 100 %.

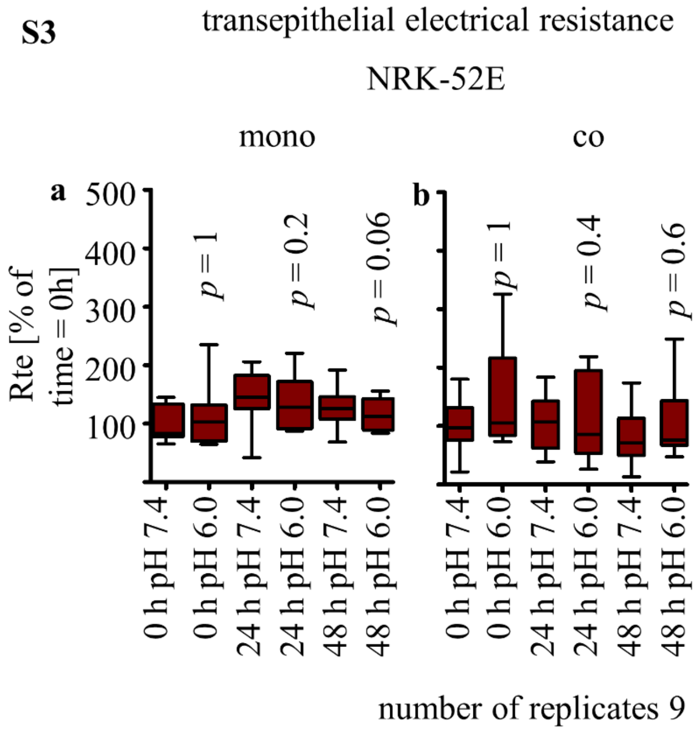

Supplementary figure S3 (S3): Impact of acidosis on TEER in NRK-52E under mono (a)- and coculture (b). $p < 0.05$ significant difference compared with the control group , n-(9). Exposure time = 48 h.

Transepithelial electric resistance (TEER) of NRK-52E cells under control conditions (pH 7.4, serum-free media for 48 h) was $128 \pm 34\ \Omega\ cm^2$ (n -(9)), a value typical for a low resistance epithelium like the proxi-mal tubule. Under acidic conditions, TEER was slightly lower compared to control conditions ($102 \pm 17\ \Omega\ cm^2$; $p = 0.06$). Acidosis did not affect TEER of NRK-52E cells under coculture conditions (fig-ure S 3b).

S4 Impact of acidosis on MMP-activity

To measure the total activity of MMPs, in cell lysates a MMP activity assay was used (Fluorometric-Red, Abcam, ab112147). A fluorescence resonance energy transfer (FRET) peptide was used as MMP sub-strate. First, pro-MMPs were activated by incubating the MMP containing samples with a 2 mM 4-Ami-nophenylmercuric Acetate (APMA) solution for 1 h at 37 ° C. Afterwards, samples were incubated with the MMP red substrate solution from the kit. The increase of fluorescence intensity as a measure for

MMP activity was determined with a fluorescence plate reader (Infinite® M200; Tecan 2.5 Männedorf, Switzerland) at Ex/Em=540/590 nm over 60 minutes (RFU/min). Finally, the results were normalized to the total cellular protein.

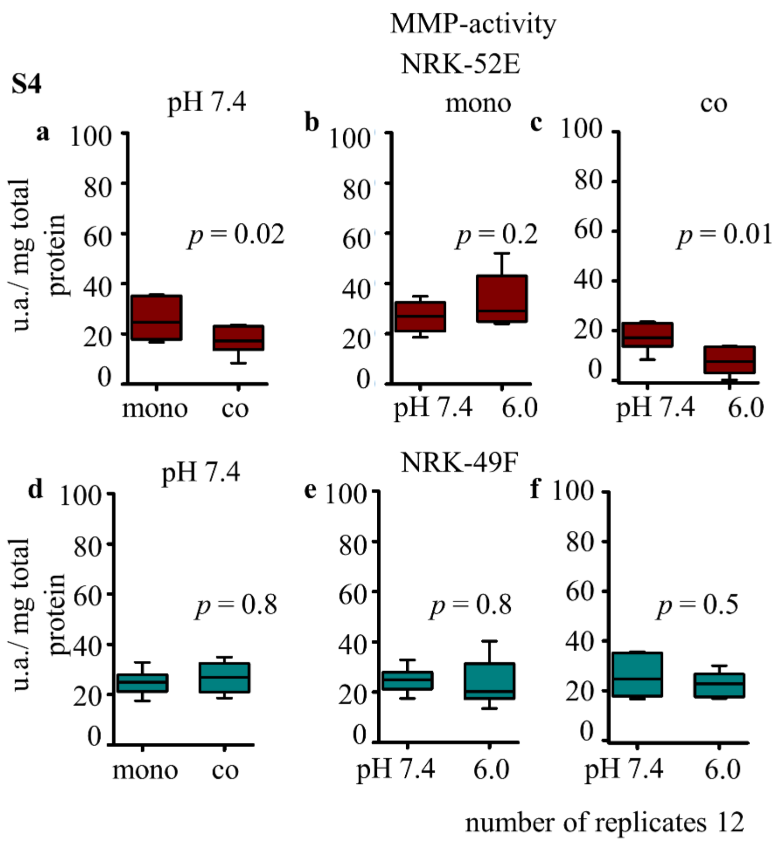

Supplementary figure S4 (S4): Impact of coculture on MMP-activity in NRK-52E (a) and NRK-49F (b). Influence of acidosis on MMP-activity in NRK-52E and NRK-49F under mono-(c, d) and coculture (e, f), p<0.05 significant difference compared with the control group, n-(9). exposure time=48 h.

Total MMP activity, determined by a FRET-peptid-based assay, was comparable in NRK-52E and NRK-49F cells. In NRK-52E cells the coculture condition led to a decrease of the MMP-activity (27±2 to 17±2; p=0.01). Additionally the incubation of NRK-52E cells in coculture, with acidic media led to a further decrease of MMP-activity (8±2; p=0.01). In NRK-49F cells neither the cultivation condition nor acidic media had an impact on MMP-activity.

S5

Impact of acidosis on mRNA expression

Supplementary Table S1 Primer sequences and annealing temperatures.

(Gene) name	Accession number	Forward 5′-3′	Backward 5′-3′	Anneal-ing tem-perature °C
Atp1b1	NM_013113	TGGA-GACTTACCCTCTGACG	GGATTTCAG-TGTCCAAGGTG	59 °C
Slc22a8	NM_031332.1	TCCTGGTGGGTAC-CAGAGTC	CTG-CATTTCTGAAGGCACAA	55 °C
Ptgs2	S67722.1	TACAAGCAGTGG-CAAAGGCC	CAGTATTGAGGA-GAACAGATGGG	60 °C
Tnf	NM_012675.3	CCACCAC-GCTCTTCTGTC-TACTGAACT	CCATTGGCCAG-GAGGGCGTT	60 °C

Abbreviations: Atp1b1: Na^+/K^+-ATPase pump; Slc22a8: organic anion transporter 3; Ptgs2: prostaglan-din-endoperoxide synthase 2; Tnf: tumor necrosis factor

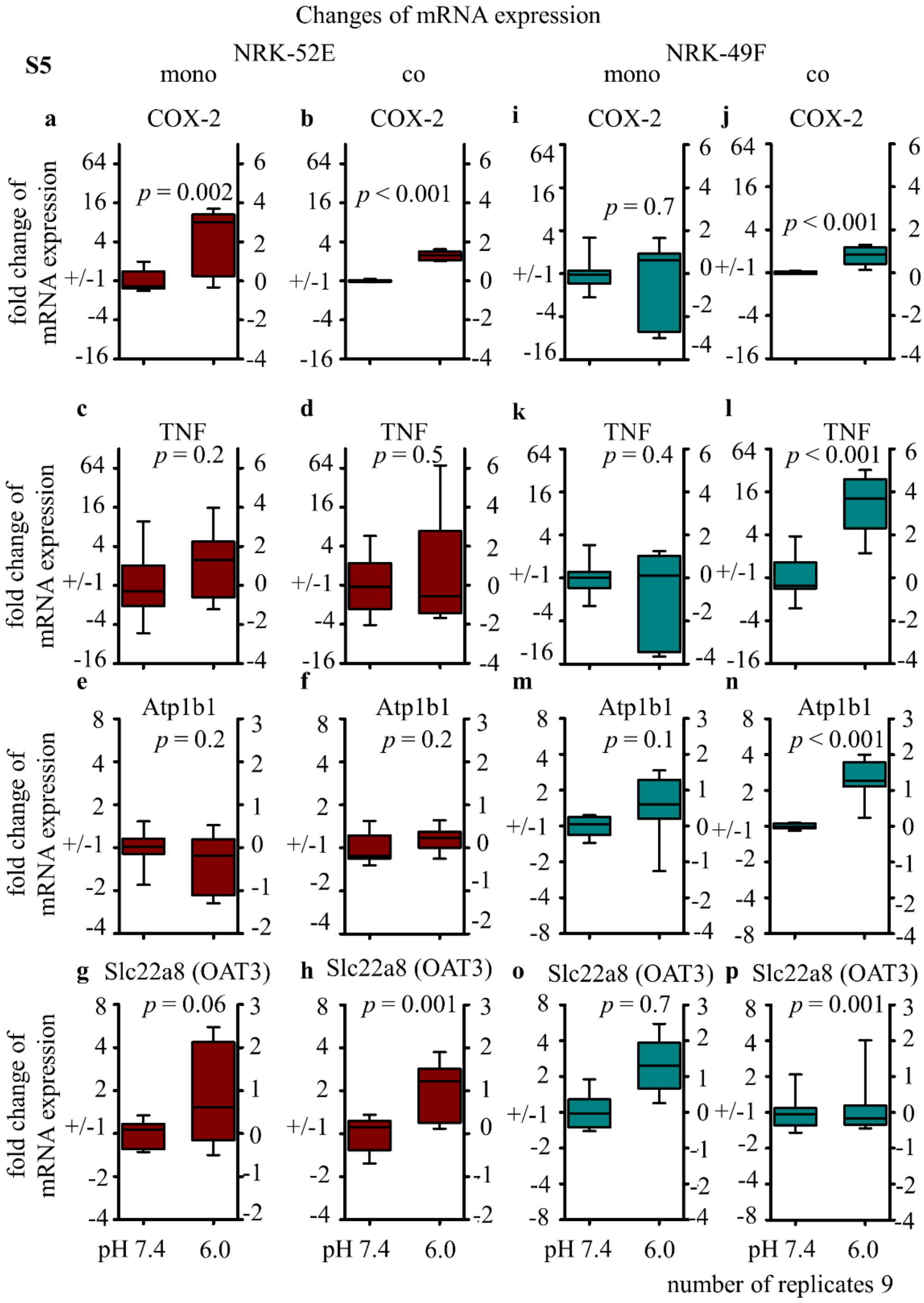

Supplementary figure S5 (S5): Influence of acidosis on mRNA expression. Impact of acidosis on COX-2 (a, b, i, j), Tnf (c, d, k, l), Atp1b1 (e, f, m, n), Slc22a8 (g, h, o, p) in NRK-52E and NRK-49F under mono and coculture. $p<0.05$ significant difference compared with the control group, n-(9). exposure time=48 h.

In NRK-52E cells in monoculture acidosis led to an increase of the COX-2 (1.2±0.4; p=0.02) mRNA-expression and a decrease of the OAT3 (-1.4±0.4; p=0.01) mRNA-expression. In coculture the acidosis-

induced increase of COX-2 (1.3±0.1; $p<0.001$) mRNA expression was still there but the OAT3 (1±0.2; $p<0.001$) mRNA expression was increased. In NRK-49F cells in monoculture acidic media led to an increase of OAT3 (1.3±0.3; $p=0.001$). In Contrast under coculture conditions this effect was vanished and the mRNA-expression of COX-2 (0.8±0.1; $p<0.001$), TNF (3.5±0.5; $p<0.001$) and Atp1b1 (1.4±0.2; $p<0.001$) was increased.

Supplementary **table S2** buffer composition

CST lysis buffer	• 20 mM TRIS; (AppliChem GmbH, Darmstadt, GER A1086,1000)<br>• 150 mM NaCl; (Carl Roth GmbH + Co. KG, Karlsruhe, GER, 3957.2)<br>• 1 % Triton X-100; (Sigma-Aldrich, Munich, GER, T-9284)<br>• 1/1000 protease inhibitor-cocktail; (Sigma-Aldrich, Munich, GER, P8340)<br>• 1 mM EDTA; (Merck KGaA, Darmstadt, GER, 1,084,180,100)<br>• 1 mM EGTA; (Merck KGaA, Darmstadt, GER, 1,084,180,100)<br>• 1 mM Na-Orthovandate; (Sigma-Aldrich, Munich, GER, S6508)<br>• 2,5 mM Na-pyrophosphate; (Sigma-Aldrich, Munich, GER, S-9515)<br>• 1 mM ß-Glycerolphosphat (Sigma-Aldrich, Munich, GER, G9422)<br>• pH adjusted to 7,5
EDTA buffer	• 136.8 mM NaCl (Carl Roth GmbH + Co. KG, GER, Karlsruhe, 3957.2)<br>• 2.68 mM KCl (Merck KGaA, Darmstadt, GER, 104936)<br>• 8.1 mM Na_2HPO_4 (Sigma-Aldrich, Munich, GER, 71645)<br>• 2 mM $H_2PO_4^-$ (Sigma-Aldrich, Munich, GER, P-6560)<br>• 0,7 mM EDTA (Merck KGaA, Darmstadt, GER, 1,084,180,100)<br>• pH7,2
ERK-lysis buffer	• 0,1 % Triton X-100 (Sigma-Aldrich, Munich, GER, T-9284) in PBS;

	• 1/1000 protease inhibitor-cocktail; (Sigma-Aldrich, Munich, GER, P8340)<br>• 0.2 mM Na_3VO_4 (Sigma-Aldrich, Munich, GER, S6508)
MOPS-Triton buffer	• 10 mM TRIS base (AppliChem GmbH, Darmstadt, GER, A1086,1000)<br>• 20 mM MOPS (Sigma-Aldrich, Munich, GER, M-3183)<br>• 100 mM NaCl (Carl Roth GmbH + Co. KG, Karlsruhe, GER, 3957.2)<br>• 1 mM EDTA (Merck KGaA, Darmstadt, GER, 1,084,180,100)<br>• 0.01 % Triton X-100 (Sigma-Aldrich, Munich, GER, T-9284)<br>• pH adjusted to 7.5
HEPES buffer	• 122.5 mM NaCl (Carl Roth GmbH + Co. KG, Karlsruhe, GER, 3957.2)<br>• 5.5 mM KCl (Merck KGaA, Darmstadt, GER, 104936)<br>• 0.8 mM $MgCl_2$ x $6H_2O$ (Sigma-Aldrich, Munich, GER, M-9272)<br>• 1.2 mM $CaCl_2$ x $2H_2O$ (Merck KGaA, Darmstadt, GER, 1.02382.1000)<br>• 1 mM NaH_2PO_4 x H_20 (Sigma-Aldrich, Munich, GER, 30186)<br>• 10 mM HEPES (Sigma-Aldrich, Munich, GER, H3034)<br>• pH7.4
6 x Redmix/Lämmli buffer	• 124.8 mM TRIS HCl (AppliChem GmbH, Darmstadt, GER, A1086,1000)<br>• 6 % SDS (Carl Roth GmbH&CoKG, Karlsruhe, GER, CN30.3)<br>• 1,42 mM ß-Mercaptoethanol (Sigma-Aldrich, Munich, GER, M3148)<br>• 4,7 mM Glycerol (Sigma-Aldrich, Munich, GER, G2025)

	• 155.2 mM Bromphenol Blue (SERVA Electrophoresis GmbH, Heidelberg, GER, 15375)<br>• pH 6.8
running buffer	• 25 mM TRIS (AppliChem GmbH, Darmstadt, GER, A1086,1000)<br>• 3,5 mM SDS (Carl Roth GmbH&CoKG, Karlsruhe, GER, CN30.3)<br>• 192 mM glycine (SERVA Electrophoresis GmbH, Heidelberg, GER, 23390.03)
transfer buffer	• 20 % MeOH (Carl Roth GmbH&CoKG, Karlsruhe, GER, T909.1)<br>• 25 mM Tris (AppliChem GmbH, Darmstadt, GER, A1086,1000)<br>• 192 mM glycine (SERVA Electrophoresis GmbH, Heidelberg, GER, 23390.03)
trypsin solution	• 154 mM NaCl (Carl Roth GmbH + Co. KG, Karlsruhe, GER, 3957.2)<br>• 2.7 mM KCl (Merck KGaA, Darmstadt, GER, 104936)<br>• 8.2 mM Na_2HPO_4 (Sigma-Aldrich, Munich, GER, 71645)<br>• 1.5 mM KH_2PO_4 (Merck KGaA, Darmstadt, GER, 1.04877.1000)<br>• 0,7 mM EDTA (Merck KGaA, Darmstadt, GER, 1,084,180,100)<br>• 0,2 mM streptomycin/0,18 mM penicillin (Sigma-Aldrich, Munich, GER, P0781)<br>• 0.02 mM trypsin (Sigma-Aldrich, Munich, GER, T0303)<br>• pH 7,1-7,3
1xTBS TWEEN	• 3 mM TRIS base (AppliChem GmbH, Darmstadt, GER, A1086,1000)<br>• 140 mM NaCl (Carl Roth GmbH + Co. KG, Karlsruhe, GER, 3957.2)

	• 0.17 mM TRIS-HCl (AppliChem GmbH, Darmstadt, GER, A1086,1000)<br>• 1 % TWEEN 20 (Sigma-Aldrich, Munich, GER, P1379)<br>• pH7.4
caspase-3 reaction buffer	• 10 mM PIPES (Sigma-Aldrich, Munich, GER, P7643)<br>• 2 mM EDTA (Merck KGaA, Darmstadt, GER, 1,084,180,100)<br>• 0.1 % CHAPS (Sigma-Aldrich, Munich, GER, C-3023)<br>• 1 % DTT (freshly added) (Sigma-Aldrich, Munich, GER, D-9779)<br>• pH7.5

1. Gekle, M., et al., Rapid activation of Na+/H+ exchange by aldosterone in renal epithelial cells requires Ca2+ and stimulation of a plasma membrane proton conductance. Proc Natl Acad Sci U S A, 1996. 93(19): p. 10500-4.
2. Gekle, M., et al., NHE3 serves as a molecular tool for cAMP-mediated regulation of receptor-mediated endocytosis. Am J Physiol Renal Physiol, 2002. 283(3): p. F549-58.
3. Thomas, J.A., et al., Intracellular pH measurements in Ehrlich ascites tumor cells utilizing spectroscopic probes generated in situ. Biochemistry, 1979. 18(11): p. 2210-8.
4. Weiner, I.D., et. al., Use of fluorescent dye BCECF to measure intracellular pH in cortical collecting tubule. Am J Physiol, 1989. 256(5 Pt 2): p. F957-64.
5. Lane, R.D., et al., Computer-assisted determination of protein concentrations from dye-binding and bicinchoninic acid protein assays performed in microtiter plates. J Immunol Methods, 1986. 92(2): p. 261-70.
